# Prognostic value of canine pancreatic lipase immunoreactivity and lipase activity in dogs with gastric dilatation-volvulus

**DOI:** 10.1371/journal.pone.0204216

**Published:** 2018-09-18

**Authors:** Giuseppe Spinella, Francesco Dondi, Lisa Grassato, Luca Magna, Veronica Cola, Massimo Giunti, Sara Del Magno, Simona Valentini

**Affiliations:** Department of Veterinary Medical Sciences, University of Bologna, Ozzano dell' Emilia (BO), Italy; Universiteit Utrecht, NETHERLANDS

## Abstract

This study evaluated the association between a selection of candidate predictor variables, including the elevation of specific pancreatic enzymes, and outcome in dogs with gastric dilatation-volvulus (GDV).

Twenty-two dogs with gastric dilatation-volvulus were included, being classified as survivors or non-survivors based on the final outcome. Dogs with intestinal obstruction (n = 16) were selected for comparison. Blood samples were collected upon admission (T0) and after 24 hours (T1). Serum lipase activity, canine pancreatic lipase immunoreactivity (cPLI) and other variables (plasma lactate concentration and C- reactive protein -CRP- in particular) were evaluated as predictive variables.

T0 cPLI and serum lipase activity were not found to differ significantly between dogs with gastric dilatation-volvulus or intestinal obstruction. Canine pancreatic lipase immunoreactivity values above 400 μg/L were detected in 6/22 dogs with gastric dilatation-volvulus and 4/16 with intestinal obstruction. However, lactate concentration was significantly higher and CRP significantly lower in GDV as compared to IO dogs, and in the GDV group, lipase, cPLI and CRP measured upon admission were significantly associated with a negative outcome. No differences in lipase activity and canine pancreatic lipase immunoreactivity values were detected between T0 and T1.

Presurgical cPLI and lipase activity were frequently increased during gastric dilatation-volvulus and were suggestive of the presence of pancreatic damage; while more extensive studies are required, based on this pilot analysis, cPLI has the potential to be a useful predictive variable for outcome in GDV. Further to this, serum CRP was able to predict outcome in this population of dogs with GDV, while blood lactate was not.

## Introduction

Gastric dilatation-volvulus (GDV) is a life-threatening condition in dogs. GDV involves acute gastric dilatation with pyloric and cardia obstruction, associated with clockwise or, less frequently, counterclockwise rotation around the mesenteric axis. [1]

GDV has a high mortality rate ranging from 10% to 43% [2–8]. Gastric wall necrosis seems related to significantly increased mortality (25–59%) [5–7, 9], and was described by Green et al. as the only variable to significantly affect survival in the population of dogs considered in their study [10].

Lipase is an enzyme produced by the pancreas, adipose tissue, gastric and duodenal mucosa with a low/moderate specificity to diagnose pancreatitis [11]. Canine pancreatic lipase is synthesized and released only by pancreatic acinar cells [12]. Canine lipase immunoreactivity is highly pancreas-specific and it has high sensitivity for pancreatitis (72%-78% in McCord et al., 2012; higher than 80% in Steiner et al., 2008). [11–14] Moreover, elevated canine pancreatic lipase immunoreactivity (cPLI) demonstrated an association with poor outcome in inflammatory bowel disease [11].

The aim of the present study was to better understand pancreatic involvement and its association with outcome in dogs with GDV. The main hypothesis was that increased cPLI concentration was associated with poor outcome in GDV and could be used as a prognostic marker; as no data regarding cPLI in GDV are currently available to perform a sample size analysis, this will be a pilot study. A secondary hypothesis that other candidate variables, such as lipase activity, plasma lactate and C-reactive protein concentration, might have predictive value was also evaluated. Finally, a comparison between GDV dogs and dogs presented with intestinal obstruction was introduced, in order to examine serum lipase activity and cPLI in animals with a different gastrointestinal tract disease and determine whether any differences were specifically caused by GDV, or were common findings in abdominal surgical diseases.

## Materials and methods

Ethical committee approval for this study was obtained at the University of Bologna, in accordance with DL 26/2014 (Project ID 581).

Presurgical data included signalment, history, clinical signs at presentation, and any administered treatment or any presurgical procedure performed at the Veterinary Teaching Hospital (VTH). Surgical medical records were used to determine: the type of surgery, the condition of the abdominal organs during the exploratory celiotomy and any complications when present. Postsurgical data included animal condition during hospitalization, the administered treatments and the final outcome. Animals receiving any medical treatment before their arrival, animals affected by pre-existing pancreatic diseases, and dogs euthanized for non-ethical reasons were excluded from this study.

GDV dogs were grouped according to their outcome: dogs that survived until discharge were classified as survivors, while those euthanized or that died spontaneously were classified as non-survivors. Euthanasia was performed in some cases with extensive gastric wall necrosis.

A control group of dogs presenting non-neoplastic intestinal obstruction (IO; n = 16) was selected. These animals were diagnosed with IO at the VTH during the same study period.

### Clinicopathological evaluation

Complete laboratory tests were performed for all the GDV dogs on admission. The serum chemistry profile (AU 480; Olympus/Beckman-Coulter) was evaluated for all patients. Blood lactate was immediately assessed using a portable lactate analyzer (Lactate Scout +, EKF diagnostics, Cardiff, UK). Blood samples were collected before starting fluid resuscitation (T0) and after 24 hours (T1) in dogs surviving to that point. Within 30 minutes after collection, these samples underwent centrifugation at 4°C, 3000 x g. After this procedure, they were immediately analyzed or stored at -80°C until the assay. Serum lipase activity (Lipase, OSR 6130, Olympus/Beckman-Coulter) was measured using the **1**,2‐diglyceride (1,2DiG) method (reference range in normal dogs: 70-700U/L); cPLI (cPLI, IDEXX Laboratories) was evaluated as well, considering values < 200 μg/L as normal, > 400 μg/L as indicative of pancreatitis, and concentrations between 200 and 400 μg/L as suspicious for pancreatitis [12]. IO dogs followed the same protocol of clinicopathological evaluation as reported for GDV dogs.

The GDV group was submitted to gastric decompression through an oral gastric tube and/or through trocarization with 18-gauge needle.

The anesthetic protocol included premedication with methadone (0.2–0.3 mg/kg IM; Synthadon, Le Vet Pharma, The Netherlands), induction with propofol (1–2 mg/kg IV; Proposure, Merial, France) and maintenance with isoflurane (Vetflurano, Virbac, France) in a mixture with pure oxygen after tracheal intubation.

All dogs were postoperatively admitted to the intensive care unit and monitored for 3–5 days after surgery.

### Statistical analysis

Data were expressed by standard descriptive statistics. Normality was tested graphically and using the D’Agostino Pearson test. Because of the non-normal distribution of most variables, non-parametric testing was adopted for all analyses and reported as median and (range). The Mann Whitney test was used to evaluate differences between groups. The Wilcoxon signed-rank test and Friedman test were conducted for comparing differences between T0 and T1. Logistic regression analyses (using univariate models) were performed to evaluate outcome prediction (stepwise approach). Variables were screened for collinearity before introduction into the regression model. Results were presented as the odds ratios (OR) and their 95% confidence intervals (CI). Overall model fit was assessed by the percentage of outcome correctly classified by the receiver operator characteristic (ROC) curve analysis and by the Hosmer & Lemeshow test (p > .05). The results of all statistics were considered significant if p ≤ .05. A sample size and power analysis was performed using the Altman’s normogram in order to determine the power of our pilot study and the number of dogs that must be included in future studies as a statistical sample to reach a minimum power of 80%. This was made on the basis of the mean values and standard deviation of cPLI, calculated as previously reported [15] in survivors and non-survivors in GDV group of this study. Statistical analyses were completed using MedCalc Statistical Software version 17.9.7 (MedCalc Software bvba, Ostend, Belgium; http://www.medcalc.org; 2017).

## Results

### Signalment

Twenty-two dogs with GDV were included in the study. The median age was 8 years (2–17) and the median body weight was 35 kg (18–55). Seventeen dogs were males (three neutered) and five were females (one spayed). Large and giant dogs predominated, accounting for 18/22 cases (seven German Shepherd Dogs or German Shepherd Dog crossbreds, four Dobermanns, two Labradors, two Bull Mastiffs, one Great Dane, one Rhodesian Ridgeback, one Neapolitan Mastiff), although 4/22 medium-sized dogs (one German Shorthaired Pointer, one Basset Hound, one French Bulldog, one Dalmatian dog) were also included.

Sixteen dogs with IO were included, with a median body weight of 16 kg (6–36) and a median age of 8.5 years (1–14). Seven of these dogs were males (one neutered) and nine were females (three spayed). This group included a wide variety of breeds (three crossbred dogs, two Boxers, two English Setters, two Labrador Retrievers, one Dachshund, one Bull Terrier, one Border Collie, one Lagotto Romagnolo, one Beagle, one Golden Retriever, one Pinscher).

### Outcome, surgical results, complications and hospitalization

Sixteen of 22 dogs (73%) with GDV survived to discharge, while 6/22 (27%) did not survive; four of them were euthanized intraoperatively due to massive gastric wall necrosis. The other two non-surviving dogs died spontaneously during the surgical procedure or a few hours later. The median age of survivors and non-surviving dogs was 8 years (3–14) and 9 years (2–17), respectively. The median body weight of survivors was 36.5 kg (18–55) while for non-survivors was 30.5 kg (24–54). Among the non-survivors, two were females and four males (one neutered).

All GDV dogs had a complete splenectomy and an incisional right gastropexy; only one of them underwent gastrotomy for the presence of a concomitant gastric acuminate foreign body that could not be removed by endoscopic procedure.

Among the IO dogs, 2/16 (12.5%) did not survive to discharge. One of them died spontaneously during a revision surgical procedure performed two days after the original enterectomy, due to extensive intestinal necrosis; while the second dog died spontaneously few hours after surgery. One of them was a neutered male and the other was a female. Their median age was 8.5 years (5–12) and their median body weight was 25 Kg (18–32). The median age and the median body weight of the surviving dogs with IO was 8.5 years (range, 1–14 years) and 14 Kg (range, 5–36.5 Kg). Eight dogs out of 16 underwent enterotomy and 3/16 dogs received enterectomy to remove intestinal foreign bodies. Two dogs out of 16 presented intestinal intussusceptions and one dog had an obstruction secondary to adhesions; in these three cases the obstruction was resolved during explorative laparotomy without any intestinal dieresis. The remaining 2/16 dogs did not need a surgical procedure as they presented a duodenal foreign body that could be removed by an endoscopic procedure.

### Laboratory results

**Fig 1:**
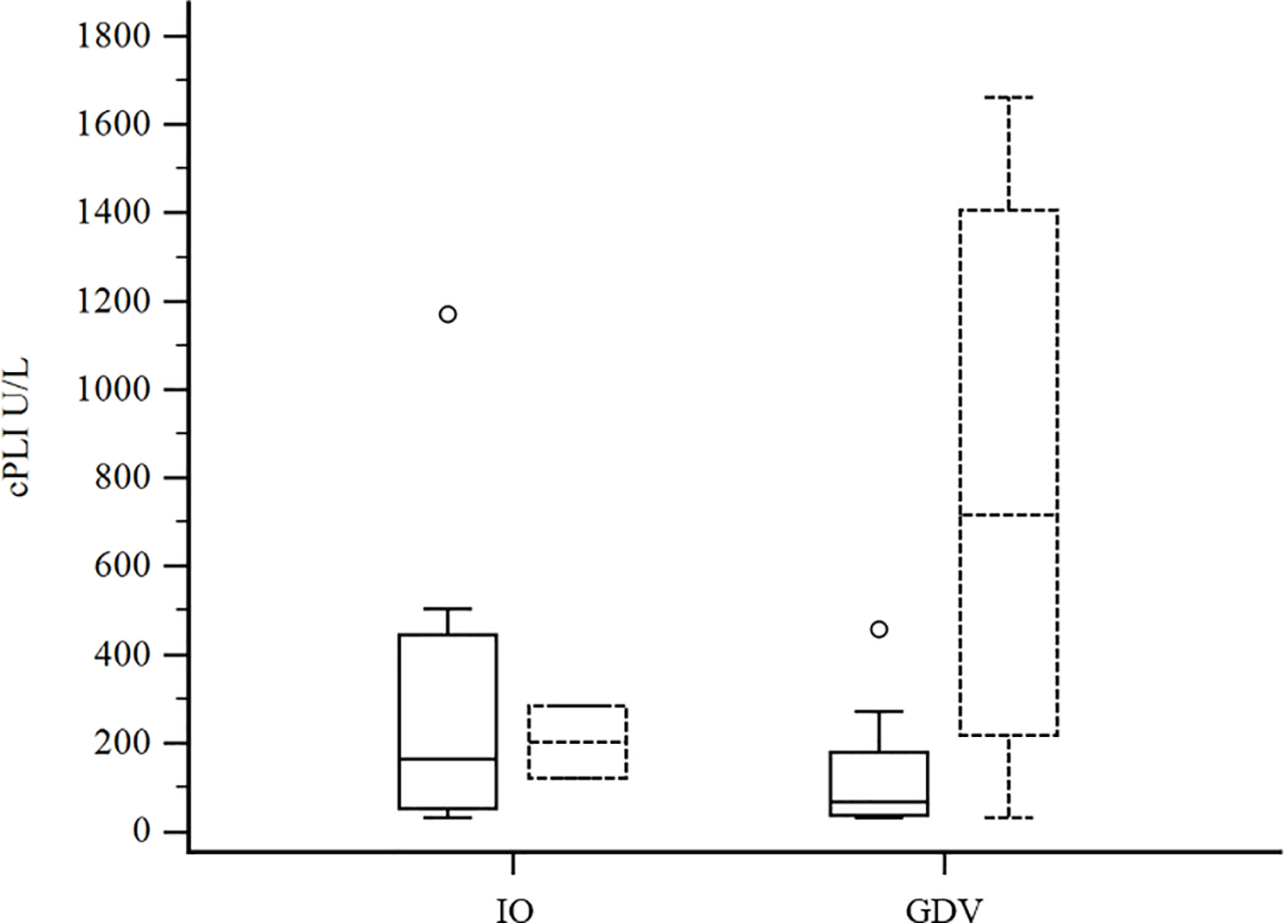
Box-and-whiskers plot of cPLI concentration (μg/L) at T0 in dogs with IO (n = 16) and GDV (n = 22). Boxes with solid lines represent survivors, while dotted lines represent non-survivors. For each box, the horizontal line represents the median value, and the upper and lower boundaries represent the 75th and 25th percentiles, respectively. Whiskers represent the minimum and maximum; circles represent outlier values.

The clinicopathological results of the GDV and IO groups at T0 are reported in Tables 1 and 2. Median cPLI concentration and lipase activity were not significantly different between GDV and IO. cPLI concentrations of survivors and non-survivors in the two groups are reported in Fig 1. Median serum lactate concentration was significantly higher in GDV group; of these 19/20 had increased serum lactate concentration (RI = 0,5–2,0 mmol/L). CRP was significantly lower in GDV dogs.

**Table 1:** Descriptive statistics and comparison among GDV and IO dogs for clinicopathological variables. Data reported as median and (range).

	Gastric dilatation-volvolus (n = 22)	Intestinal obstruction (n = 16)
**Lactate (mmol/L)**	5.55 (1.8–12.1) ^a^	1.65 (0.7–9.6)
**cPLI (**μg **/L)**	101 (30–1784)	163 (30–1170)
**Lipase (U/L)**	260 (37–1106)	247 (38–1009)
**CRP (mg/dL)**	1.71 (0.01–9.89) ^a^	7.3 (0.18–27.04)

^a^ significantly different from IO dogs (p≤0.05)

cPLI = canine pancreatic lipase immunoreactivity; CRP = C-reactive protein.

**Table 2:** Descriptive statistics of surviving and non-surviving dogs for clinicopathological variables. Data reported as median and (range).

	Gastric dilatation-volvolus		Intestinal obstruction	
	Survivors (n = 16)	Non-survivors (n = 6)	Survivors (n = 14)	Non-survivors (n = 2)
**Lactate (mmol/L)**	5.25 (1.8–10.30)	7.1 (3.1–12.1)	1.7 (0.7–9.6)	1.2 (0.8–1.6)
**cPLI (**μg **/L)**	67 (30–1784)	716.5 (30–1661)	163 (30–1170)	201.5 (119–284)
**Lipase (U/L)**	237 (37–1080)	582.5 (38–1106)	233 (38–1009)	359 (247–471)
**CRP (mg/dL)**	1.05 (0.01_9.47)	5.79 (2.03–9.89)	6.42 (0.18–27.04)	7.89 (7.3–8.48)

In GDV group, lipase, cPLI and CRP were significantly associated with a negative outcome, while lactate concentration was not; Logistic regression analysis results were reported in Table 3.

**Table 3:** Results of the univariate logistic regression analyses for the prediction of mortality in dogs with GDV. Only variables with p < 0.2 are reported.

**Univariate logistic regression**			
**Variable**	OR	95% CI	p value
**cPLI (U/L)**	1.0017	0.9998–1.0037	.**0501**
**Lipase (U/L)**	1.0035	1.0001–1.0070	**.0443**
**Lactate (mmol/L)**	1.3417	0.9324–1.9307	.1134
**CRP (mg/dL)**	1.4204	1.0080–2.0016	.**049**

cPLI = canine pancreatic lipase immunoreactivity; CRP = C-reactive protein

Based on ROC curve analysis, cPLI had poor accuracy for predicting outcome. Instead CRP, with a cutoff of 2,02 mg/dL, had 100% sensitivity and 80% specificity for prediction of outcome (AUC = 0,850).

No differences in cPLI values were detected between T0 and T1 in the GDV dogs (p = 0.95), with a median value less than the lower threshold for both. cPLI values did not differ between GDV and IO dogs both at T0 (p = 0.7) and at T1 (p = 0.85).

Furthermore, neither the cPLI values (p = 0.6 for IO), nor lipase activity differed significantly between T0 and T1 in the two pathological groups (p = 0.4 and p = 0.6 for IO and GDV respectively).

Finally, considering the absolute difference of 294 μg/L of the mean values between survivors and non-survivors in our sample of 22 GDV dogs, this study appears to have a 40% power to detect that difference with statistical significance.

In order to achieve a minimum of 80% power in a future assessment of cPLI as an outcome predictor in GDV, we calculate that a sample of at least 78 dogs is required assuming a similar mortality rate.

## Discussion

The high mortality rate related to GDV motivates the investigation of biomarkers that could provide a prognostic aid to the veterinary surgeon. Our results showed that cPLI concentration was higher in non-surviving GDV dogs (median 716 μg/L) than in survivors (median 67 μg/L), although this difference was not significant (p = 0.08).

Previously, the myoglobin level at the time of GDV diagnosis (Mbt0) has been found to predict outcome, but with only moderate sensitivity. Adamik et al. reported that 90% of dogs presenting a Mbt0 < 168 ng/mL (considered the cutoff value) survived, while 50% of dogs with Mbt0 > 168 ng/mL did not survive [16]. Similarly, presurgical serum pepsinogen-A concentration was significantly associated with gastric wall lesion severity, but it turned out to be only a moderate prognostic factor [17]. Coagulation parameters and inflammatory markers do not seem to have significant prognostic value in GDV dogs [17, 18]; on the contrary, in this study, an increase in CRP concentration was significantly associated with a worse outcome. This discrepancy indicates the need for further studies with larger sample populations in order to understand the real importance of acute-phase proteins as an outcome-specific predictor. Plasma lactate concentration and plasma lactate clearance during resuscitation are the most studied and commonly used parameters in clinical practice [6, 10, 19, 20], although their usefulness is not completely understood. Israeli et al. argued that lactate concentration is mostly affected by the severity of systemic hypoperfusion, acidosis and shock, and that it is only indirectly associated with the presence and extent of gastric wall necrosis [17]. Zacher et al. and Green et al. reported that there was no significant correlation between hyperlactatemia at presentation and gastric wall necrosis or outcome [6, 10]; this is consistent with the results of the present study, in which most of the dogs presented hyperlactatemia but blood lactate concentrations did not appear to be associated with outcome.

Our decision to investigate lipase and cPLI serum concentrations as prognostic factors came from the study of the results of previous works evaluating these parameters before and after surgery [17, 21]. Pancreatitis is a possible complication in dogs with GDV: it seems to derive from gastric and splenic displacement [17, 21] or from the decreased venous return which could cause hypoperfusion, resulting in pancreas inflammation [22].

Lipase is considered to have low/moderate accuracy and specificity (60%) for diagnosing pancreatitis in dogs [11, 23, 24]. Simpson et al. demonstrated the presence of serum lipase activity in pancreatectomized dogs [25], while dogs presenting exocrine pancreatitis but with serum lipase concentrations within the reference range were reported by Steiner et al. [26]. Moreover, it has been found that non-pancreatic pathologies (i.e. renal failure, intestinal diseases, hepatic diseases) could cause alterations in lipase activity [27, 28], while 47.6% of 70 dogs with fatal pancreatitis presented normal values of lipase serum concentration [29].

In contrast, cPLI is synthesized and released only by pancreatic acinar cells, and its immunoreactivity is pancreas-specific [12]. In the veterinary literature, cPLI values higher than the threshold of 400 μg/L are considered to have a very high specificity for the diagnosis of pancreatitis (ranging between 97.5 and 100%). [12, 30]

Israeli et al. reported an increased serum cPLI value of over 200 μg/L in 58% of dogs with GDV included in their study, with 18% of these animals recording a cPLI concentration higher than 400 μg/L [17]. In the present study approximately 36% of dogs had an increased cPLI concentration, and dogs with cPLI values above the cutoff value of 400 μg/L accounted for 27% of the study population.

A previous study investigated the role of cPLI as a prognostic factor in dogs affected by inflammatory bowel disease, highlighting that there is a negative correlation between cPLI values and outcome [11]. Our results could indicate that the pancreas is likely to be affected by inflammation during gastrointestinal tract pathologies, with consequent cPLI alterations, without a specific correlation with GDV (lipase activity and cPLI values did not differ significantly between GDV and IO dogs both at T0 and at T1).

Another hypothesis that supported indirect secondary pancreatic activation was proposed by Li et al. in 2013 and investigated through experiments on rats. The authors observed a noteworthy level of “neural intimacy” between the pancreas and duodenum [31]. This finding was a direct effect of an indirect vagal pathway that is common to most upper gastrointestinal organs and suggests that an acute lesion to one of these organs could lead to indirect stimulation of others [31]. If this finding was replicated in dogs, it would provide an easy explanation of the increased cPLI in dogs with GDV, without macroscopic pancreatic disease or alteration.

An interesting result of the present study is that lipase activity and cPLI values were correlated with outcomes in GDV dogs, confirming our main hypothesis. As already stated, the involvement of an organ different from pancreas could cause an increase in lipase activity and this extended organ damage could explain its association with outcome. However, the correlation between cPLI and outcome was borderline significant (p = 0.05): dogs with a higher concentration of cPLI were more likely to have a bad outcome, a result that encourages further studies of this relationship in a larger sample of GDV dogs.

GDV dogs included in the present study demonstrated neither classical clinical signs of pancreatitis, nor macroscopic signs of inflammation on direct visual examination of the pancreas during surgery, or during the ultrasound examinations. This is in line with the study by Israeli et al., in which only one dog presented clinical signs of pancreatitis postoperatively, recording the highest serum cPLI concentration among the dogs included in that cohort (3380 μg/L) [17]. These findings indicate that pancreatic damage may occur during GDV, but without impacting the clinical condition of the dogs. However, it is also possible that the symptoms of pancreatitis were masked by the postoperative treatment of GDV, which is almost identical to the management of pancreatitis.

A further point of note is that, in a recent study, a high agreement was found between cPLI and lipase activity measured using a 2-o-dilauryl-rac-glycero-3-glutaricacid-(6′-methylresorufin) ester (DGGR) assay [32]. In the present work, a 1,2 DiG method was used for the lipase measurements, but evaluation of lipase activity via DGGR in dogs presenting GDV has never been performed and could provide useful additional results.

This study had several limitations. A small number of GDV and IO dogs were included, limiting the strength of the statistical analyses as indicated by the performed power analysis. Moreover, pancreatic biopsies were not performed and so histological data, the gold standard for diagnosis of pancreatitis, were not available to compare to lipase and cPLI values.

In conclusion, in this population of dogs with GDV, serum CRP was able to predict outcome while blood lactate was not. Presurgical cPLI concentration and lipase activity are frequently increased in abdominal surgical diseases, and particularly in GDV. Based on our pilot analysis, cPLI has the potential to be a useful predictive variable for outcome in GDV. To confirm this in a potential future prospective study, assuming the same mortality risk, a sample size of at least 78 dogs would be recommended.
